# Supplementary material for: The causal role of circulating inflammatory markers in osteoporosis: a bidirectional Mendelian randomized study
Source: Front Immunol. 2024 Jul 18;15:1412298. doi: 10.3389/fimmu.2024.1412298 (PMC11291241; doi:10.3389/fimmu.2024.1412298)
Supplement: Supplementary file 1 [file DataSheet_1.zip › supplementary material/OSTEOPOROSIS-CIM/GCST90274778.tsv.gz_leave_one_out.pdf]

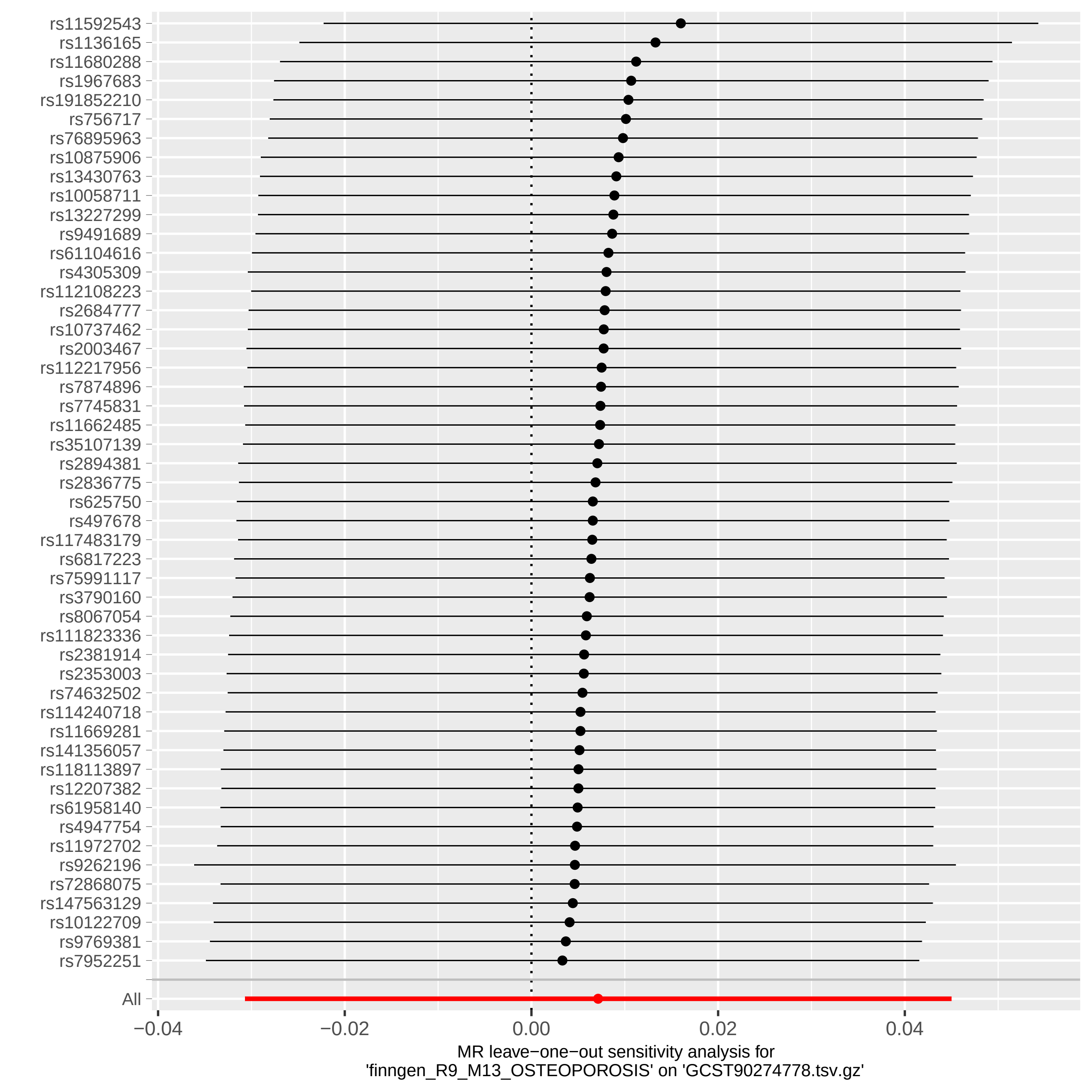
rs11592543
rs1136165
rs11680288
rs1967683
rs191852210
rs756717
rs76895963
rs10875906
rs13430763
rs10058711
rs13227299
rs9491689
rs61104616
rs4305309
rs112108223
rs2684777
rs10737462
rs2003467
rs112217956
rs7874896
rs7745831
rs11662485
rs35107139
rs2894381
rs2836775
rs625750
rs497678
rs117483179
rs6817223
rs75991117
rs3790160
rs8067054
rs111823336
rs2381914
rs2353003
rs74632502
rs114240718
rs11669281
rs141356057
rs118113897
rs12207382
rs61958140
rs4947754
rs11972702
rs9262196
rs72868075
rs147563129
rs10122709
rs9769381
rs7952251
All
−0.04
−0.02
0.00
0.02
0.04
MR leave−one−out sensitivity analysis for
'finngen_R9_M13_OSTEOPOROSIS' on 'GCST90274778.tsv.gz'
